# Obesity and Gastrointestinal Diseases

**DOI:** 10.1155/2013/760574

**Published:** 2013-05-27

**Authors:** Ai Fujimoto, Shu Hoteya, Toshiro Iizuka, Osamu Ogawa, Toshifumi Mitani, Yuichiro Kuroki, Akira Matsui, Masanori Nakamura, Daisuke Kikuchi, Satoshi Yamashita, Tsukasa Furuhata, Akihiro Yamada, Noriko Nishida, Koji Arase, Mitsuyo Hashimoto, Yoshinori Igarashi, Mitsuru Kaise

**Affiliations:** ^1^Department of Gastroenterology, Toranomon Hospital, 2-2-2, Toranomon, Minato-ku, Tokyo 105-8470, Japan; ^2^Department of Gastroenterology and Hepatology, Toho University, Omori Medical Center, 6-11-1 Omorinishi, Otaku, Tokyo 143-8541, Japan

## Abstract

The prevalence of obesity in the Japanese population has been increasing dramatically in step with the Westernization of lifestyles and food ways. Our study demonstrated significant associations between obesity and a number of gastrointestinal disorders in a large sample population in Japan. We demonstrated that reflux esophagitis and hiatal hernia were strongly related to obesity (BMI > 25) in the Japanese. In particular, obesity with young male was a high risk for these diseases. On the other hand, it has been reported that obesity is also associated with Barrett's esophagus and colorectal adenoma; however, obesity was not a risk factor for these diseases in our study. The difference of ethnicity of our subjects may partly explain why we found no data to implicate obesity as a risk factor for Barrett's esophagus. Arterial sclerosis associated with advanced age and hyperglycemia was accompanied by an increased risk of colorectal adenoma.

## 1. Introduction

The prevalence of obesity in the Japanese population has been increasing dramatically in step with the Westernization of lifestyles and foodways [1]. Obesity is primary among the medical disorders composing metabolic syndrome and is associated with many gastrointestinal diseases. Among the upper gastrointestinal diseases, obesity is an established risk factor for reflux esophagitis [2]. As the obese population grows, so too grows the incidence of reflux esophagitis, a condition strongly linked to obesity. According to a retrospective analysis of the endoscopic findings of 23,870 Japanese patients, the incidence of reflux esophagitis rose from 1% in 1975–1977 to 2% in 1995–1997 [3]. In a prospective study conducted in Japan more recently, reflux esophagitis was detected in as many as 16% of the subjects [4].

Reflux esophagitis is considered to be a risk factor for Barrett's esophagus [5–8], a condition found in about 10% of patients diagnosed with reflux esophagitis [9, 10]. Obesity is established to be a strong risk factor for reflux esophagitis. While this suggests that obesity may have a direct or indirect association with Barrett's esophagus, no definitive studies have been done to confirm this.

Among lower gastrointestinal diseases, obesity is consistently related to an elevated risk of colon cancer [11]. The metabolic syndrome, in turn, is associated with an elevated risk of colorectal adenoma [12]. The association of obesity with diverticula of the colon is controversial. Aldoori et al. reported a weak correlation between elevated body mass index (BMI) and symptomatic diverticular disease of colon [13], whereas other authors found this correlation to be absent [14–16].

In the present study we sought to elucidate the associations between obesity and various upper and lower gastrointestinal diseases in a large sample of Japanese subjects.

## 2. Methods

### 2.1. Subjects

This study was retrospectively conducted at the Healthcare Administration Center of Toranomon Hospital with a population of 42,862 Japanese adults who underwent health check-ups between January 2008 and December 2010. We analyzed subjects who underwent upper gastrointestinal endoscopy and/or colonoscopy during routine health check-ups. After excluding subjects with prior gastric or colon surgery, 18,792 consecutive individuals who underwent upper gastrointestinal endoscopy (mean age 54.2 ± 10.9, male : female ratio 13310 : 5482) and 1,586 consecutive patients who underwent colonoscopy (mean age 56.6 ± 10.7, male : female 1245 : 341) were eligible.

The upper gastrointestinal endoscopies and colonoscopies were performed by endoscopists working in the Gastroenterology Department of Toranomon Hospital. All of the examiners had more than 5 years of experience in endoscopy and were blinded to the results of the blood test screenings, physical and physiological examinations, and questionnaires.

### 2.2. Definitions and Classifications of Obesity and Various Gastrointestinal Disorders

The definition of obesity was based on a report from the Japan Society for the Study of Obesity. In the classification by BMI, subjects with a BMI of 24.9 kg/m^2^ or less were normal and those with a BMI of 25.0 kg/m^2^ or more were obese [17].

The classification of reflux esophagitis used in the endoscopic recording system of this institute was based on the Los Angeles classification proposed at the World Congress of Gastroenterology in 1994. The presence of lesions graded M or higher under the LA classification was adopted as an inclusion criterion for reflux esophagitis for this study [18].

Barrett's esophagus was defined as abnormal columnar esophageal epithelium suggestive of a columnar epithelium-linked distal esophagus under macroscopic observation [19].

Hiatal hernia was diagnosed when the distance between the gastroesophageal junction and the diaphragmatic hiatus was 2 cm or more [20].

### 2.3. Definition of Hypertension

Hypertension was defined if the subjects were diagnosed with hypertension and were currently under treatment or follow up for the condition.

### 2.4. Classification of Alcohol Consumption and Smoking History

The validity of the information on alcohol consumption and smoking history was confirmed independently by interviews with both a doctor and nurse for each subject. Smoking histories were classified into four ordinal groups according to the Brinkman index (cigarettes per day multiplied by years of smoking): nonsmoker, 0; light smoker, 1–200; moderate smoker, 200–400; and heavy smoker, >400 [21]. The total amount of alcohol consumed per week (volume per day multiplied by drinking days per week) was calculated in grams and categorized into four grades: nondrinker, <40 g/week; light drinker, 40–140 g/week; moderate drinker, 140–280 g/week; and heavy drinker, >280 g/week. The alcohol content of each beverage mentioned in the questionnaire was as follows: beer (5% alcohol by volume), Japanese sake (brewed from rice; 15% alcohol by volume), and Japanese shochu (distilled from sweet potatoes, rice, or buckwheat; 25% alcohol by volume).

### 2.5. Statistical Analysis

We investigated whether the following diseases were more prevalent in the obese group than in the nonobese group: reflux esophagitis, hiatal hernia, Barrett's esophagus, gastric ulcer or gastric ulcer scar, duodenal ulcer or duodenal ulcer scar (upper gastrointestinal disease), and colorectal adenoma and diverticula (lower gastrointestinal disease). Differences in the prevalence of gastroenterological disease between the obese group and nonobese group were tested using the *χ* ^2^ test. Next, a multiple logistic regression analysis of the following independent variables was performed to determine significant risk factors for the diseases found to be significantly more prevalent in the obese group: gender, age, obesity (BMI > 25), hemoglobin A1c (HgbA1c), triglyceride (TG), total cholesterol (TCHO), hypertension, smoking habit, and drinking habit.

Values are expressed as means with standard deviations (SDs) or as percentages. Odds ratios (ORs) and 95% confidence intervals (CIs) were determined for each variable. A *P* value of less than 0.05 was considered statistically significant. All analyses were performed using Stat View version 5.0 (SAS Institute Inc., Cary, NC, USA).

## 3. Results

### 3.1. Characteristics of Enrolled Subjects

Over the test period covered, 42,862 Japanese adults underwent routine health check-ups at our hospital. Of these subjects, 18,792 (mean age 54.2 ± 10.9, male : female ratio 13310 : 5482) who underwent upper gastrointestinal endoscopy and 1,586 (mean age 56.6 ± 10.7, male : female 1245 : 341) who underwent colonoscopy were eligible after exclusion.

In total, 4,415 (23.4%) of the 18,792 patients who underwent upper gastrointestinal endoscopy and 456 (28.7%) of the 1,586 who underwent colonoscopy met the Japanese criteria for obesity.

Among the 18,792 subjects who underwent upper gastrointestinal endoscopy, 4,355 (23.1%) were diagnosed with reflux esophagitis. The numbers and rates of hiatal hernia, Barrett's esophagus, gastric ulcer (scar), and duodenal ulcer (scar) were 4,731 (25.1%), 1,492 (7.9%), 158 (0.8%), and 1,353 (7.2%), respectively. Among the 1,586 subjects who underwent colonoscopy, 495 (31.2%) and 182 (11.4%) were diagnosed with colorectal adenomas and diverticula of the colon, respectively.

### 3.2. Prevalence of Gastrointestinal Disease in Obese versus Nonobese Groups

Compared with the nonobese subjects, significantly higher proportions of obese subjects were diagnosed with reflux gastritis (32.3% versus 20.3%, *P* < 0.0001), hiatal hernia (33.1% versus 22.7%, *P* < 0.0001), Barrett's esophagus (9.2% versus 7.5%, *P* = 0.0004), and colorectal adenoma (36.8% versus 28.9%, *P* = 0.0023). There were no significant differences between these groups in the proportions of subjects with gastric ulcers (or scars), duodenal ulcers (or scars), or diverticula of the colon (Table 1).

### 3.3. Predictors for Gastrointestinal Disease

Multivariate analysis showed that male gender (ORs = 2.02; 95% CIs = 1.83–2.23; *P* ≤ 0.0001), younger age (ORs = 0.99; 95% CIs = 0.98-0.99, *P* ≤ 0.0001), hypertriglyceridemia (ORs = 1.001; 95% CIs = 1.001-1.002; *P* ≤ 0.0001), obesity (ORs = 1.51; 95% CIs = 1.40–1.64; *P* ≤ 0.0001), hypertension (ORs = 1.11; 95% CIs = 1.01–1.21; *P* = 0.02), and heavy drinker status (ORs = 1.30; 95% CIs = 1.17–1.44; *P* ≤ 0.0001) were independent risk factors for reflux esophagitis (Table 2). Likewise, male gender (ORs = 2.008; 95% CIs = 1.82–2.20; *P* ≤ 0.0001), young age (ORs = 0.99; 95% CIs = 0.992–0.998; *P* = 0.0042), hypertriglyceridemia (ORs = 1.001; 95% CIs = 1.000-1.001; *P* = 0.0001), and obesity (ORs = 1.41; 95% CIs = 1.30–1.52; *P* ≤ 0.0001) were independent risk factors for hiatal hernia (Table 3). Male gender (ORs = 1.93; 95% CIs = 1.66–2.26; *P* < 0.0001), age (ORs = 1.005; 95% CIs = 0.99–1.01; *P* = 0.07), reflux esophagitis (ORs = 1.32; 95% CIs = 1.171–1.488; *P* < 0.0001), and hiatal hernia (ORs = 3.68; 95% CIs = 3.29–4.12; *P* < 0.0001) were risk factors for Barrett's esophagus. Obesity was not identified as an independent risk factor for Barrett's esophagus (Table 4).

The odds ratios for male gender and obesity were higher in patients suffering from both reflux esophagitis and hiatal hernia than in patients suffering from only one of those disorders (male: ORs = 2.49; 95% CIs = 2.14–2.90; *P* ≤ 0.0001; obesity: ORs = 1.76; 95% CIs = 1.58–1.95; *P* ≤ 0.0001) (Table 5). Male gender (ORs = 1.78; 95% CIs = 1.27–2.49; *P* = 0.0008), old age (ORs = 1.03; 95% CIs = 1.01–1.04; *P* ≤ 0.0001), hyperglycemia (ORs = 1.28; 95% CIs; 1.07–1.52; *P* = 0.0059), hypertriglyceridemia (ORs = 1.003; 95% CIs; 1.001–1.005; *P* = 0.0013), and heavy drinker status (ORs = 1.84; 95% CIs = 1.32–2.56; *P* = 0.0003) were independent risk factors for colorectal adenoma, while obesity was not (Table 6).

In a multivariate analysis of women only, obesity was the sole risk factor for reflux esophagitis (ORs = 1.90, 95% CIs: 1.52–1.90, *P* ≤ 0.0001), while old age (ORs = 1.01, 95% CIs: 1.003–1.018, *P* = 0.0079), hypertriglyceridemia (ORs = 1.002, 95% CIs: 1.001–1.004, *P* = 0.0048), obesity (ORs = 2.287, 95% CIs: 1.865–2.804, *P* ≤ 0.0001), and heavy smoker status (ORs = 1.919, 95% CIs: 1.057–3.486, *P* = 0.0322) were all risk factors for hiatal hernia.

## 4. Discussion

The present study demonstrated significant associations between obesity and a number of gastrointestinal disorders in a large-sample population. Obesity was an independent risk factor for both reflux gastritis and hiatal hernia. It was also found to be a stronger risk factor for patients who had both disorders concurrently than for patients who had only one. By widespread consensus, obesity-associated reflux esophagitis is thought to develop when excess abdominal fat mechanically increases the gastric pressure and thereby reduces the frequency of esophageal sphincter relaxation with acid reflux [22–27]. Yet the mechanisms by which obesity affects reflux esophagitis remain partly controversial. The increase in abdominal pressure by obesity is reportedly too moderate to induce reflux in experimental models [25, 28–30]. Others have hypothesized that visceral adipose tissue contributes to gastrointestinal disorders. Visceral adipose tissue is recognized to be metabolically active and has been strongly associated with elevated serum levels of proinflammatory adipokines that may contribute to the development of reflux gastritis, namely, interleukin 6, tumor necrosis factor-*α*, and adiponectin [26, 31]. The precise mechanisms by which obesity induces gastric acid reflux need to be elucidated by further studies.

 Young males were at higher risk for both reflux esophagitis and hiatal hernia, whereas the multivariate analysis identified advanced age as a risk factor for hiatal hernia in women. The presumed mechanism for hiatal hernia and reflux esophagitis in young obese males is increased abdominal pressure, while the presumed mechanism for hiatal hernia in older females is thoracic kyphosis secondary to osteoporosis [32]. The mechanism of hiatal hernia apparently differs among genders and generations.

Though linked to reflux esophagitis and hiatal hernia, obesity was not a significant risk factor for Barrett's esophagus in our study. Most previous studies have confirmed obesity, gastroesophageal reflux, male gender, Caucasian ethnicity, and increased age as the established risk factors for Barrett's esophagus [33]. The difference of ethnicity of our subjects may partly explain why we found no data to implicate obesity as a risk factor for Barrett's esophagus. Several studies using patients of different racial groups examined in the same endoscopy units demonstrated that the prevalence of Barrett's esophagus was lower in Asians than in Caucasians [34]. The risk factors for Barrett's esophagus may differ from one ethnicity to another. A previous Japanese study showed that older age, male gender, and hiatal hernia were all risk factors for Barrett's esophagus, while obesity was not [35]. Further studies on ethnic differences in the “Barrett's gene,” parietal cell mass, hiatal hernia, *H. pylori*, and inflammatory mediators may further elucidate the reasons for the interethnic variations in Barrett's esophagus observed.

Obesity is also consistently related to an increased risk of colon cancer [11]. There were no cases of colon cancer in our study. Our findings did show, however, that arterial sclerosis associated with advanced age and hyperglycemia was accompanied by an increased risk of colorectal adenoma, while obesity was not. Visceral fat leads to a high influx of abundant free fatty acids and proinflammatory cytokines into the liver, which in turn accentuates insulin resistance and a local and systematic proinflammatory process [36]. Insulin may exert a proliferative effect on colonic tumor cells directly [37, 38]. Obesity and insulin resistance associated with visceral fat may be stronger risk factors for colon adenoma than simple obesity.

Different ethnicities also show variation in diabetes mellitus and BMI categories. The prevalence of diabetes mellitus in Asians in a BMI range of 22.0–24.9 was comparable to that in Caucasians in a BMI range as high as 34.0–36.0 [39]. When we set a BMI of 23.5 as a cut-off with our data, a BMI beyond the cutoff was a risk factor for colorectal adenoma. (*P* = 0.023, ORs = 2.25, 95% CI: 1.03–1.84.) A significant association between BMI and colorectal adenomas with a dose-response relationship was observed in a population of Korean subjects, but the analysis was performed at a cutoff lower than BMI 25, the presumed cutoff for Asians [40].

If the prevalence of obesity continues to rise as dramatically as it has been as a consequence of globalization and the Westernization of Asian countries, the association between obesity and colorectal adenoma may grow remarkably, as well. 

## 5. Conclusion

In summary, obesity is strong risk factor for reflux gastritis and hiatal hernia, even when affected by other factors.

## Figures and Tables

**Table 1:** Prevalence of gastrointestinal disease in obese versus non-obese groups.

Upper gastrointestinal disease (*n* = 18792)	Obesity group (*n* = 4415)	Nonobesity group (*n* = 14377)	*P* value
RE (*n* = 4355)	1427 (32.3%)	2928 (20.3%)	<0.0001
Hiatal hernia (*n* = 4731)	1465 (33.1%)	3266 (22.7%)	<0.0001
Barrett's esophagus (*n* = 1492)	407 (9.2%)	1085 (7.5%)	0.0004
Gastric ulcer and/or gastric ulcer scar (*n* = 158)	40 (0.9%)	118 (0.8%)	0.5727
Duodenal ulcer and/or duodenal ulcer scar (*n* = 1353)	313 (7.0%)	1040 (7.2%)	0.7646

Lower gastrointestinal disease (*n* = 1586)	Obesity group (*n* = 456)	Non-obesity group (*n* = 1130)	*P* value

Colorectal adenoma (*n* = 495)	168 (36.8%)	327 (28.9%)	0.0023
Diverticula of colon (*n* = 182)	60 (13.1%)	122 (10.7%)	0.1919

**Table 2:** Predictors for reflux esophagitis.

	Odds ratio	95% CI	*P* value
Sex: male	2.02	1.83–2.23	<0.0001
Age (years)	0.99	0.98–0.99	<0.0001
HgbA1c (%)	1.02	0.96–1.08	0.46
TG (mg/dL)	1.001	1.001–1.002	<0.0001
TCHO (mg/dL)	1.001	0.99–1.001	0.38
Obesity: BMI > 25 kg/m^2^	1.51	1.40–1.64	<0.0001
Hypertension	1.11	1.01–1.21	0.02
Heavy drinker	1.30	1.17–1.44	<0.0001
Heavy smoker	0.89	0.79–1.01	0.08

HgbA1c: hemoglobin A1c.

TG: triglyceride.

TCHO: total cholesterol.

BI: Brinkman index.

**Table 3:** Predictors for hiatal hernia.

	Odds ratio	95% CI	*P* value
Sex: male	2.008	1.82–2.20	<0.0001
Age (years)	0.99	0.992–0.998	0.0042
HgbA1c (%)	1.004	0.94–1.06	0.89
TG (mg/dL)	1.001	1.000–1.001	0.0001
TCHO (mg/dL)	1.001	1.000–1.002	0.20
Obesity: BMI > 25 kg/m^2^	1.41	1.30–1.52	<0.0001
Hypertension	1.06	0.97–1.15	0.18
Heavy drinker	1.02	0.92–1.13	0.68
Heavy smoker	1.06	0.94–1.19	0.29

**Table 4:** Predictors for Barrett's esophagus.

	Odds ratio	95% CI	*P* value
Sex: male	1.93	1.66–2.26	<0.0001
Age (years)	1.005	0.99–1.01	0.07
HgbA1c (%)	0.98	0.89–1.08	0.74
TG (mg/dL)	1.000	1.000–1.001	0.15
TCHO (mg/dL)	1.001	1.000–1.003	0.10
Obesity: BMI > 25 kg/m^2^	1.08	0.95–1.23	0.20
Hypertension	1.02	0.88–1.17	0.77
Heavy drinker	0.96	0.82–1.12	0.63
Heavy smoker	0.25	0.92–1.32	1.11
Reflux esophagitis	1.32	1.17–1.48	<0.0001
Hiatal hernia	3.68	3.29–4.12	<0.0001

**Table 5:** Predictors for patients suffering from both reflux esophagitis and hiatal hernia.

	Odds ratio	95% CI	*P* value
Sex: male	2.49	1.24–2.90	<0.0001
Age (years)	0.99	0.990–0.999	0.027
HgbA1c (%)	1.007	0.92–1.09	0.87
TG (mg/dL)	1.001	1.001–1.002	<0.0001
TCHO (mg/dL)	1.001	0.99–1.002	0.50
Obesity: BMI > 25 kg/m^2^	1.76	1.58–1.95	<0.0001
Hypertension	1.04	0.92–1.18	0.43
Heavy drinker	1.27	1.10–1.46	0.0001
Heavy smoker	0.88	0.75–1.05	0.16

**Table 6:** Predictors for colorectal adenoma.

	Odds ratio	95% CI	*P* value
Sex: male	1.78	1.27–2.49	0.008
Age (years)	1.03	1.01–1.04	<0.0001
HgbA1c (%)	1.28	1.07–1.52	0.0059
TG (mg/dL)	1.003	1.001–1.005	0.0013
TCHO (mg/dL)	1.001	0.99–1.005	0.53
Obesity: BMI (kg/m^2^)	1.15	0.89–1.48	0.25
Hypertension	1.03	0.78–1.35	0.83
Heavy drinker	1.84	1.32–2.56	0.0003
Heavy smoker	0.85	0.59–1.22	0.38
